# Correction: Habitat complexity influences neuron number in six species of Puerto Rican *Anolis*

**DOI:** 10.1098/rsbl.2025.0193

**Published:** 2025-06-04

**Authors:** Levi Storks, Jessica Garcia, Christian A. Perez-Martinez, Manuel Leal

**Affiliations:** ^1^ Division of Biological Sciences, University of Missouri, Columbia, MO, USA; ^2^ Department of Biology, University of Detroit Mercy, Detroit, MI, USA; ^3^ College of Veterinary Medicine, University of Minnesota, Minneapolis, MN, USA

*Biol. Lett.* **20**, 20230419 (Published 7 February 2024). (https://doi.org/10.1098/rsbl.2023.0419)

In the originally published version of this article [1], an error occurred in the conversion from grams to milligrams when reporting data on the mass of each brain region. Specifically, in table 1, the values for mean region mass and their associated standard errors were transcribed as one order of magnitude smaller than observed values. The error affected only the mean region mass column in table 1.

**Table 1.** Summarized data by species and brain region. Ecomorph classification is indicated under species name. Means are presented ± standard error. RHC, relevant habitat complexity; SVL, snout vent length; *n,* sample size; *N,* Neuron number.

species	RHC	mean SVL (cm)	mean mass_Body_ (g)	brain region	*n*	mean *N* x 10^6^	mean mass_Region_ (mg)
*A. cristatellus* trunk–ground	2.6196	5.5 ± 0.088	4.6 ± 0.24	telencephalon	14	1.88 ± 0.0885	17.7 ± 0.681
				ROB	14	2.26 ± 0.0907	28.1 ± 0.744
				cerebellum	13	0.84 ± 0.0547	1.9 ± 0.1
*A. evermanni* trunk–crown	−5.1598	5.6 ± 0.2	4.4 ± 0.41	telencephalon	10	1.73 ± 0.08	15.6 ± 1.03
				ROB	10	2.62 ± 0.128	26.4 ± 1.68
				cerebellum	8	0.619 ± 0.0622	1.7 ± 0.12
*A. gundlachi* trunk–ground	8.1306	6 ± 0.075	4.9 ± 0.24	telencephalon	10	1.59 ± 0.144	18.5 ± 0.776
				ROB	10	2.06 ± 0.0784	30.8 ± 0.835
				cerebellum	10	0.65 ± 0.0741	2.4 ± 0.12
*A. krugi* grass–bush	3.2436	4.8 ± 0.097	2.5 ± 0.19	telencephalon	10	1.37 ± 0.101	13.3 ± 0.603
				ROB	9	1.9 ± 0.132	21 ± 0.664
				cerebellum	10	0.532 ± 0.0787	1.5 ± 0.075
*A. pulchellus* grass–bush	−0.3601	4.4 ± 0.12	1.7 ± 0.14	telencephalon	10	1.24 ± 0.0512	9.14 ± 0.489
				ROB	10	1.79 ± 0.0635	15.9 ± 0.713
				cerebellum	10	0.522 ± 0.0452	1.2 ± 0.076
*A. stratulus* trunk–crown	−8.3974	4.3 ± 0.11	1.6 ± 0.11	telencephalon	10	1.61 ± 0.11	11.1 ± 0.248
				ROB	10	2.22 ± 0.0943	18 ± 0.629
				cerebellum	10	0.705 ± 0.0578	1.4 ± 0.097

A related error occurred in our data analysis that impacted the output datasheet included as electronic supplementary material (rsbl20230419_si_003.csv). This resulted in density values to be reported as three orders of magnitude smaller than observed values due to a double conversion error. The affected columns were: *CellDensity_cell.Mg, DensityN_N.mg, DensityNonN_NonN.mg, logDensityN_N.Mg* and *logDensityNonN_NonN.mg*. All other data in this spreadsheet were accurate. Below is an updated table 1, and corrected versions of the supplementary material data (rsbl20230419_si_003.csv) and analysis (rsbl20230419_si_002.RMD) files are available online [2].

These errors had no effect on the results of this study, including those presented in the Results section or figure 1. As detailed in the Methods section and shown in the accompanying analysis files, the data used in the MCMC analyses and to generate figure 1 did not rely on the incorrect density values. We apologize for any confusion or inconvenience these errors may have caused.
